# RNA binding proteins as mediators of pathological cardiac remodeling

**DOI:** 10.3389/fcell.2024.1368097

**Published:** 2024-05-16

**Authors:** Pooja Acharya, Sharon Parkins, Michael Tranter

**Affiliations:** ^1^ Department of Molecular Medicine and Therapeutics, The Ohio State University Wexner Medical Center, Columbus, OH, United States; ^2^ Dorothy M. Davis Heart and Lung Research Institute, The Ohio State University Wexner Medical Center, Columbus, OH, United States; ^3^ Department of Internal Medicine, Division of Cardiovascular Health and Disease, University of Cincinnati College of Medicine, Cincinnati, OH, United States

**Keywords:** RNA binding proteins (RBPs), post-transcriptional gene regulation, cardiac, hypertrophy, fibrosis

## Abstract

RNA binding proteins (RBPs) play a central in the post-transcriptional regulation of gene expression, which can account for up to 50% of all variations in protein expression within a cell. Following their binding to target RNAs, RBPs most typically confer changes in gene expression through modulation of alternative spicing, RNA stabilization/degradation, or ribosome loading/translation rate. All of these post-transcriptional regulatory processes have been shown to play a functional role in pathological cardiac remodeling, and a growing body of evidence is beginning to identify the mechanistic contribution of individual RBPs and their cardiac RNA targets. This review highlights the mechanisms of RBP-dependent post-transcriptional gene regulation in cardiomyocytes and fibroblasts and our current understanding of how RNA binding proteins functionally contribute to pathological cardiac remodeling.

## Introduction

An emerging body of literature is establishing a central role for RNA binding proteins (RBPs) in modulating pathological cardiac remodeling (Neumann et al., 2022; Robinson and Port, 2022; Kelaini et al., 2021; D’Antonio, et al., 2022). Approximately 3,000 genes in the human genome code for RNA binding proteins, and the diversity and functional distribution of many of the known RBPs has been previously reviewed in depth (Gerstberger et al., 2014). RBPs execute their regulatory functions by forming ribonucleoprotein complexes (or interactomes) by interacting with RNA in a dynamic and combinatorial manner. These interactomes may also contain RNA modifying enzymes which can influence RNA-protein interactions and ultimately drive RBP-dependent regulation of RNA stability, alternative polyadenylation and splicing, subcellular localization, and translation of mature mRNAs by ribosomes (Park et al., 2011; Gilbert et al., 2016; de Bruin et al., 2017; Hoernes and Erlacher, 2017; Hentze et al., 2018; Cornelius et al., 2022).

The cellular and molecular mechanisms of pathological cardiac remodeling encompass cardiomyocyte hypertrophy and enhanced fibroblast extracellular matrix (ECM) remodeling activity that ultimately lead to functional contractile deficiencies (Mishra and Kass, 2021; Chen and Peng, 2023; Guo et al., 2023). Fibroblasts comprise approximately 10%–20% of the total cell population in a healthy heart, but their proliferation, activation to a myofibroblast state, and increased ECM remodeling drives cardiac fibrosis, which is a ubiquitous hallmark of the failing heart. The activation of quiescent cardiac fibroblasts to pro-fibrotic myofibroblasts can occur in response to multiple stimuli including TGFβ, Wnt, Angiotensin II, or mechanotransduction (DeLeon-Pennell et al., 2020; Gibb Andrew et al., 2020). Regardless of the activating stimulus, cell differentiation to a myofibroblast and the resulting ECM remodeling activity are dependent on large scale transcriptomic and proteomic changes.

RBPs play a critical role in post-transcriptional regulation and the dynamic expression of both the transcriptome and proteome through RNA transcript-specific localization, stability, and translation. For example, it was recently demonstrated that roughly one-third of all TGFβ-dependent gene expression changes in cardiac fibroblasts are subject to translational regulation independent of RNA expression (Chothani et al., 2019). However, transcriptomic and proteomic analyses are rarely done in parallel, which can lead to key post-transcriptional regulatory processes being overlooked. This review will summarize the current knowledge of the field with regard to how RBP-dependent post-transcriptional gene regulation contributes to pathological cardiac remodeling, with a specific focus on their actions in cardiac myoyctes and fibroblasts.

To understand the mechanisms of actions of RBPs, it is essential to also briefly review the post-transcriptional processes they control. These mechanisms, which can be simplified into the three primary categories of alternative splicing, RNA stability/degradation, and translation, have been reviewed in depth elsewhere, but we will provide an overview here with a discussion of how they have been shown to impact pathological cardiac remodeling along with the individual RBPs that utilize these functions to modulate the cardiac transcriptome.

### Alternative splicing

Pre-mRNAs are composed of introns and exons which can be included or excluded in a biologically regulated manner from the final mature mRNA sequence leading to multiple alternatively spliced transcripts (Sharp, 2005). Alternative splicing of these transcripts not only contributes to mRNA function and diversity, but also has downstream effects on RBP-dependent binding and functional modulation of the RNA transcript (Kelemen et al., 2013). Alternative splicing, mediated by several different RBPs, including RBM20/24, ASF/SF2, SRSF, CUGBP/CELF, MBNL, and Rbfox family members, has an established role in cardiac development and homeostasis as well as in response to pathological stimuli (Xu et al., 2005; Kalsotra et al., 2008; Kalsotra et al., 2010; Koshelev et al., 2010; Dasgupta and Ladd, 2012; Guo et al., 2012; Linke and Bucker, 2012; Blech-Hermoni and Ladd, 2013; Li et al., 2013; Wei et al., 2015; Gao et al., 2016). The best example of the importance of alternative splicing in cardiac physiology may be in the expression diversity of titin, one of the most abundant proteins in cardiomyocytes and a key component of the contractile sarcomere. Titin exists as a single gene with all expression variation manifesting as a result of alternative splicing, that is responsible for a developmental shift in titin isoform expression from fetal to adult hearts. Alternative splice variants of titin have been shown to confer different mechanical stiffness to the sarcomeres that directly impact contractility, and splicing mutants have been implicated in hereditary cardiomyopathies (LeWinter and Granzier, 2014).

#### RNA binding motif (RBM) proteins

Dozens of RBM protein family members have been annotated and shown to regulate RNA metabolism through splicing, stability, and translation in multiple tumor types (Li et al., 2010). However, RBM20 and RBM24 have been most studied in the heart as they have been shown to govern the alternative splicing of titin, and deletion of RBM20 results in the exclusive expression of the longer, fetal isoform of titin that impairs cardiac contractility in adult hearts (Guo et al., 2012). Unsurprisingly, mutations in RBM20 have been associated with dilated cardiomyopathies in humans (Brauch et al., 2009; Li et al., 2010). RBM20 has been shown to control the ratio of titin isoforms in rats following exercise, with exercise being associated be associated with an enriched expression ratio of a titin splice variant shown to be more mechanically compliant (Chung et al., 2020).

In addition to titin, RBM20 controls the splicing fates of crucial cardiac genes such as CamkIIδ, and RyR2, such that RBM20 gene mutation causes mis-splicing events of these cardiac genes, resulting in early onset dilated cardiomyopathy (Guo et al., 2012; van den Hoogenhof et al., 2018). The post-transcriptional processes controlled by RBM20 also extend to beyond alternative splicing of target RNA. For instance, RBM20 has been suggested to be at the forefront of ribonucleoprotein (RNP) granule regulation as well. RNP granules are intracellular condensates composed of RNA and protein, held together in a dynamic manner (Shin and Brangwynne, 2017; Schneider et al., 2020). The functional role of RNP granules are mostly unknown as they are akin to membrane-less organelles that comprise high localized RNP concentrations and are still being investigated (Ripin and Parker, 2023). However, RBM20 deficiency leads to dysregulated RNP granules that potentially drives myocardial pathobiology and heart failure (Schneider et al., 2020). A mutation in RBM20 has been identified to have a causal role in cardiac splicing alterations and re-distributes ribonucleoprotein granules within processing bodies (Fenix et al., 2021).

Similarly, RBM24, has also been shown to affect the alternative splicing of titin as well as other z-disc proteins in the sarcomere such as Nebl, Ablim1, and Enah (Liu et al., 2019). Knockdown of RBM24 also decreased the expression of genes encoding components of the contractile machinery and energy production (Liu et al., 2019). Rbm24 has been extensively assessed in zebrafish and adeno-associated virus (AAV9)-mediated RBM24 over the expressing mouse models (Poon et al., 2012; van den Hoogenhof et al., 2018). The findings in the zebrafish model reveal that RBM mutations can lead to sarcomere-related cardiomyopathy via regulation of sarcomere assembly and cardiomyocyte contractility (Poon et al., 2012). Overexpression of RBM24 can have a deleterious effect by inducing cardiac fibrosis, as seen through Rbm24-dependent splicing differences in cardiac genes such as PDZ and Lim domain 5, phospholamban, and Titin. This was accompanied with robust periostin expression, indicating Rbm24 mediates regulation of cardiac fibrosis, potentially via the regulation of TgfβR1 and TgfβR2 expression (van den Hoogenhof et al., 2018).

#### RNA binding Fox-1 homologs (Rbfox)

The Rbfox proteins, a conserved RBP family of alternative splicing mediators whose expression is enriched in both skeletal and cardiac muscle, has been suggested to play a role in both cardiac development and homeostatic maintenance in adult cardiomyocytes (Gallagher et al., 2011; Cao et al., 2021; Huang et al., 2022; Verma et al., 2022). Wei et al. (2015) reported decreased expression of Rbfox2 in mouse hearts in response to pressure overload (TAC)-induced heart failure and that cardiomyocyte-specific deletion of Rbfox2 is sufficient to induce contractile dysfunction and heart failure. Other groups have suggested that loss of Rbfox2 splicing function is an early pathological mediator in diabetic cardiomyopathy (Nutter et al., 2016). However, the mechanisms for this remain unclear as Rbfox2 has been shown to have potential splicing-independent roles as well and may contribute to pathological cardiac remodeling through transcriptional modulation of miRNAs (Hu et al., 2019).

Gao et al. (2016) also showed decreased cardiac expression of Rbfox1 in failing human hearts as well in mouse model of pressure overload-induced heart failure. They went on to show that Rbfox has a widespread effect on cardiac splice events during pathological progression, but Rbfox-mediated alternative splicing of MEF2, a key transcriptional regulator of hypertrophic gene expression in cardiomyocytes, plays a particularly important role. Importantly, they also showed that restoring Rbfox1 expression specifically in cardiomyocytes ameliorated pressure overload-induced cardiac dysfunction and pathology. Both Rbfox1 and two have also been suggested to contribute to myocyte hypertrophy and progression to heart failure through alternative splicing of the Ca_V_1.2 L-type calcium channels (Wang et al., 2018; Li et al., 2023). Mutations in both Rbfox1 and Rbfox2 have also been linked to cardiac pathology in humans, but continued work is needed to fully understand the regulation of RNA targeting and functional contribution of Rbfox-mediated splice variants (Lale et al., 2011; Verma et al., 2016).

#### Muscleblind-like 1 (MBNL1)

MBNL1 has been suggested as a master regulator of the transformation of fibroblasts to myofibroblasts, which is a key step for wound healing and fibrotic remodeling, through direct binding and regulating a network of differentiation-specific and cytoskeletal/matrix-assembly transcripts which aids in myofibroblast differentiation (Davis et al., 2015; Stempien-Otero et al., 2016; Bugg et al., 2022). MBNL1 expression in the myocardium increases significantly within 4 days of myocardial infarction or in response to TGFβ-dependent stimulation of fibroblasts *in vitro*. Functionally, MBNL1 expression in fibroblasts appears to be dependent on their differentiation to myofibroblasts. Further, numerous MBNL1 target transcripts were identified in active myofibroblasts that play a role in several cell signaling pathways. For instance, mutations in MBNL1 lead to selective modulation of its target transcripts Calcineurin Aß, serum response factor (SRF), and p38 which can lead to fibrosis (Davis et al., 2015; Stempien-Otero et al., 2016). Furthermore, fibroblast state plasticity is also determined by MBNL1 as a post-transcriptional process which influences cardiac wound healing (Bugg et al., 2022). The pivotal regulatory position of MBNL1 in the myofibroblast differentiation signaling network makes it an attractive target to novel RNA-based therapeutic strategies, to slow down fibrosis and scarring during disease (Davis et al., 2015).

Additional work is needed to conclusively define the cell-type specific function of MBNL1 in cardiac remodeling, and MBNL1 may play an equally important role in cardiomyocytes. During early developmental stages, MBNL1 is a critical regulator of cardiomyocyte cell cycle entry and proliferation through altered cell cycle inhibitor transcript stability. MBNL1 is suggested to function as a transcriptome-wide switch between regenerative and mature myocyte states postnatally and throughout adulthood (Bailey et al., 2023). Deletion of MBNL1 results in the expression of multiple embryonic splice variants within the cardiac transcriptome, including genes regulating sodium and calcium currents as well as structural components of the sarcomere and cytoskeleton (Dixon et al., 2015). Many of these MBNL1-dependent splice variants, along with MBNL1 itself, have also been implicated in the disease progression of myotonic dystrophy type I (DM1), for which heart disease and sudden cardiac death are prominent causes of mortality (Philips et al., 1998). At the molecular level, DM1 is caused by expansion and expression of CUG repeats that accumulate and sequester MBNL proteins in the nucleus, reducing their native splicing activity (Miller et al., 2000; Fardaei et al., 2002; Mankodi et al., 2005). It was recently demonstrated that cardiomyocyte-specific deletion of both MBNL1 and MBNL2 recapitulates the cardiac pathology of DM1 and results in cardiac hypertrophy, fibrosis, and an increased prevalence of sudden cardiac death (Lee et al., 2022).

#### CUG-BP and ETR-3-like factors (CELF)

The alternative splicing function of CELF family proteins, of which CUG-BP1 and ETR-3 are the two most highly expressed in the heart, play a critical role in cardiac development. Cardiomyocyte-specific expression of a truncated dominant negative CELF protein was shown to specifically disrupt CELF-dependent splicing and leads to the development of cardiac hypertrophy and fibrosis in young (3–9 week-old) mice when expressed *in vivo* (Ladd et al., 2005a; Ladd et al., 2005b). However, the same group went on to show that this CELF-dependent cardiomyopathy was spontaneously overcome without intervention as the mice aged, with no remaining deficits in cardiac function detectable at 24 weeks of age in mice with a mild expression of the CELF dominant negative protein (Terenzi et al., 2009). Interestingly, CELF-dependent splice events were still found to be suppressed following functional recovery, suggesting either a threshold of CELF function required for homeostasis in the adult heart or a CELF-independent recovery of cardiac function in the adult heart. Indeed, the expression of CUG-BP1 and ETR-3 are both highest at birth and decrease with age, supporting a strong role for both in cardiac development. However, their functional role has not been investigated in the setting of cardiac pathology, but these results would suggest that they might play a key role in mediating expression of the fetal gene program that is associated with myocyte hypertrophy in the adult heart. The postnatal expression of MBNL and Fox proteins have been shown to increase as CELF protein expression decreases, and this transition in splicing protein expression pattern may regulate more than half of the development splicing pattern during cardiac development (Kalsotra et al., 2008; Gazzara et al., 2017).

#### Serine/arginine-rich splicing factor (SRSF) proteins

There are 12 SRSF family members (SRSF1-12), are traditional nuclear splicing factors that have also been shown to shuttle to the cytoplasm where thay can also regulate mRNA stability and translation (Twyffells et al., 2011). Deletion of SRSF1/2 or 10 disrupted postnatal and embryonic cardiac remodeling, in part through a dysregulation of contractility mediated by alternative splicing of CamKII and triadin, respectively, suggesting a role for these RBPs in cardiac development (Ding et al., 2004; Xu et al., 2005; Feng et al., 2009). In the adult heart, SRSF3 expression was shown to be reduced following ischemic injury, and its deletion in cardiomyocytes induces rapid (within 8 days) systolic failure and death that was contributed to alterations in mTOR splicing and subsequent mRNA decapping and reduced translation of contractile and calcium handling genes (Ortiz-Sanchez et al., 2019).

Alternative splicing as a posttranscriptional mechanism regulated by RBPs in fibroblasts is not limited to pathology, but also plays a role in the reprogramming of cells in the adult heart based on environmental cues. The involvement of RBPs in various cell lineages and pathways of cardiac reprogramming have also been studied using single cell genomics. These studies delineate that the downregulation of the RBP polypyrimidine tract binding protein (PTBP) allows cell transitions between cardiac fibroblasts (CFs) to induced cardiomyocytes (iCMs) (Liu et al., 2017). Accordingly, PTBP has been shown to play a role in cardiac development by its expression in both cardiomyocytes and endothelial cells through regulating the translation of apoptotic genes (Apaf-1 and Caspase-3) and splicing of β-arrestin-1, respectively (Zhang et al., 2009; Liu et al., 2023).

Additional RBPs are suggested to modulate cardiac physiology through splicing regulation, but with less understood about their mechanisms or targets in the heart. For example, RNA binding protein with multiple splicing (RBPMS), which significantly impacts myofibrillar organization and calcium handling through alternative splicing of regulators such as titin, Pdlim5 and nexilin (Akerberg et al., 2022; Gan et al., 2022). Similarly, the Quaking (QKI) family of RBPs function as alternative splicing factors of sarcomere and cytoskeletal component genes, calcium-handling genes, and post-transcriptional regulators (Kelaini et al., 2021; Cornelius et al., 2022). QKI isoforms themselves are products of alternative splicing with roles in angiogenesis, cell migration and adhesion senescence (Montañés-Agudo et al., 2023).

RBPs involved in mRNA processing and alternative splicing can also act as a molecular roadblock in generating the post-transcriptional splicing patterns needed to reprogram mouse fibroblasts into induced cardiomyocytes (Liu et al., 2017; Ricketts and Qian, 2022). Most investigations focus extensively on gene regulatory networks determining fibroblast transcriptomic fates, while overlooking the post-transcriptional regulation that controls the proteomic landscape. RBPs are at the forefront of the post-transcriptional regulation and have the potential to serve as a therapeutic target as their modulation can trigger changes in several genes in any cell type and disease model (Bretherton et al., 2020).

In addition to alternative splicing, eukaryotic RNA can also be regulated through alternative polyadenylation (APA) which can generate multiple variants of the same RNA with distinct 3′UTR sequence lengths (Tian and Manley, 2017). APA is highly prevalent in that 50%–70% of transcripts encoded by human genes are estimated to contain multiple alternative poly (A) sites in their 3′UTR (Tian et al., 2005; Derti et al., 2012). APA sites generate multiple mRNA transcripts, thereby altering mRNA coding potential; changing availability of RBP and microRNA binding sites, and thus determining mRNA fates (Di Giammartino et al., 2011). RBP mediated regulation of APA can occur by either directly constituting the cleavage and polyadenylation machinery or inhibiting the APA process or binding through adjacent region of the target poly (A) sites (Zheng and Tian, 2014).

The specific impact of APA on heart development and its dysregulation in cardiac pathology has been reviewed elsewhere (Cao and Kuyumcu-Martinez, 2023). mRNA isoform and microarray analyses of hypertrophic mouse hearts reveal genome-wide APA changes, wherein more than half of the 315 tandem APA events led to 3′UTR shortening. This was accompanied by embryonic-like APA patterns in the hypertrophic hearts, considering fetal gene expression is a hallmark of cardiac hypertrophy (Park et al., 2011; Cao and Kuyumcu-Martinez, 2023). Interestingly, most cardiac RBPs that are actively involved in alternative splicing, also display function as APA regulators. For instance, importance of MBNL1 in APA has been demonstrated through HITS-CLIP and minigene reporter analyses, and the loss of MBNL in mouse embryonic fibroblasts has been shown to dysregulate a lot of AP events (Batra et al., 2014; Davis et al., 2015). Many other important RBPs such as human antigen R (HuR) and PTBP, have also been implicated for their APA modulating activities on their target mRNA transcripts. HuR and PTB regulate cardioprotective genes like heat shock protein (HSP70.3) and cyclooxygenase (COX-2), respectively by interacting with the upstream sequence elements in the 3′-UTR that control the efficiency of polyadenylation (Tranter et al., 2011; Kraynik et al., 2015; Hall-Pogar et al., 2007).

### RNA stability/degradation

Another key step modulated predominately by RBPs is control of mRNA stability, which directly determines the half-life of individual transcripts within the cells and thus impacts both the availability and capacity for translation of mRNAs, but also the function of other RNAs, such as circular or long non-coding RNAs (lncRNAs). lncRNA have themselves emerged as key modulators of mRNA stability, which can act either independently or synergistically with RBPs (Sebastian-delaCruz et al., 2021). mRNA stability is broadly determined by the combined actions of specific sequences within the RNA itself (*cis*-acting elements), typically within the 3′-untranslated region (UTR), and the interacting RBPs (*trans*-acting factors) that drive the stabilizing and destabilizing actions. These interactions occur in a cell type and disease dependent manner and are dependent on the expression and activation profile of RBPs as well as the transcriptome of available binding targets (Wu and Brewer, 2012). For instance, in the muscles of heart and other tissues, expression of sarcoplasmic reticulum calcium-ATPase 2 (SERCA2a) is influenced by RBPs that can have a destabilizing (e.g., AUF1) or stabilizing effect (e.g., HuR) on the mRNA stability, and even small changes in SERCA can result in direct functional effects on Ca^2+^ and ß-adrenergic signaling within contractile cells (Misquitta et al., 2006). Furthermore, the posttranscriptional regulation of G protein-coupled β adrenergic receptors (β-AR) in cardiomyocytes was also demonstrated to occur at the level of mRNA stability (Eberhardt et al., 2007). In addition, mRNA stability can also be heavily impacted by the degrading actions of miRNAs, such as miR-21, which has shown to be a major effector of cardiac fibrosis (Thum et al., 2008; Piccoli et al., 2016).

Many RBPs, such as ARE/poly(U)-binding/degradation factor 1 (AUF) and Human antigen R (HuR) have been demonstrated to regulate mRNA stability and degradation through specific binding to AU-rich elements (AREs), which are most typically found in the 3′untranslated region (3′UTR) of mRNAs (Gingerich et al., 2004). These AREs often form short RNA hairpin loops that facilitate RBP recognition through both a sequence and structural manner. AUF1 is a prominent example of an RBP that operates through ARE binding and subsequent recruitment of members of the mRNA degradation machinery. HuR on the other hand tends to promote stability of target transcripts following ARE recognition and binding (Eberhardt et al., 2007; Schultz et al., 2020). The balance of this regulation is dependent on expression, localization, and post-translational regulation of individual RBPs as well as the expression and relative stoichiometry of potentially available ARE-containing RNA targets. Additionally, RBPs may compete with RNA-RNA driven regulation of target transcripts, such as those mediated by lncRNA or miRNA (Engreitz et al., 2014; Xue, 2022). To this end, both HuR and AUF1 have been shown to compete with miRNA binding for mRNA target recognition and have even been shown to modulate the expression of some miRNAs (Srikantan et al., 2012). Much of this is governed by the pathophysiological state of the tissue and cell types, and while both HuR and AUF1 have been shown to play a role in the heart (discussed in detail in subsequent sections), the details of how these processes play out across distinct cell populations of the myocardium to mediate pathological cardiac remodeling remains poorly understood.

#### Human antigen R (HuR)

HuR is a nearly ubiquitously expressed RNA binding protein whose cardiac expression was first shown to be increased at 3 days post-MI (Krishnamurthy et al., 2010). Inhibition of HuR in the post-ischemic heart was subsequently shown to reduce pathological remodeling through suppression of inflammatory signaling, with a potential direct regulation of TGFβ and p53 mRNA stability (Krishnamurthy et al., 2010; Slone et al., 2023).

Our lab has shown that HuR is both necessary and sufficient for hypertrophic growth of cardiac myocytes (Slone et al., 2016). We have also shown that HuR expression and cytoplasmic translocation, an indicator of RNA binding activity, is increased in failing human hearts and that both cardiomyocyte-specific ablation or pharmacological inhibition of HuR reduces the progression of cardiac hypertrophy and fibrosis in a pressure overload (transverse aortic constriction; TAC) model of heart failure (Green et al., 2019). HuR has also been suggested to regulate the RNA stability of sodium channel (SCN5A) and calcium cycling modulator (phospholamban) genes during cardiac remodeling (Zhou et al., 2018; Hu et al., 2020).

In addition to its role in cardiomyocytes, we found HuR to be highly expressed in cardiac fibroblasts, and necessary for their pro-fibrotic response and phenotypic transformation to myofibroblasts (Green et al., 2023). Interestingly, our previous work in cardiomyocytes also identified a HuR-dependent regulation of TGFβ as well as secreted pro-inflammatory cytokines, suggesting that HuR may also orchestrate myoycte-centric signaling to other cardiac cell types (Green et al., 2019; Slone et al., 2023). To this end, Govindappa et al. (2020) showed that HuR-dependent extracellular vesicle (EV) signaling from macrophages may mediate fibroblast activity and fibrosis in diabetes associated cardiac remodeling. These results are consistent with other work showing HuR-dependent regulation of endocrine signaling from other cell types, including the modulation of extracellular vesicle secretion (Deng et al., 2009; Fabbiano, et al., 2020; Mukherjee, et al., 2016; Shi, et al., 2020; Deng, X., et al., 2020), and our additional work showing an adipose tissue-derived HuR-dependent endocrine impact on cardiac remodeling (Guarnieri et al., 2021).

#### ARE/polyU binding/degradation factor1 (AUF1)

AUF1, as the name suggests, is an RNA binding protein that binds to AU rich or poly U regions of mRNA and selects that RNA for rapid degradation. AUF1 itself does not have the ability to degrade the RNA, but the binding of AUF1 coordinates the recruitment and complex assembly of other trans-acting proteins to form the RNA degradation machinery. AUF1 expression was shown to be increased in failing human hearts, likely downstream of β-adrenergic receptor (β-AR) signaling (Pende et al., 1996). They also showed that AUF1 binds to the β_1_-AR mRNA and postulate that AUF1 contributes to the downregulation of β_1_-AR mRNA in cardiomyocytes observed in failing hearts (Pende et al., 1996).

AUF1 expression was shown to increase in response to angiotensin II (AngII) in cardiomyocytes to mediate the hypertrophic downregulation of myocyte voltage-gated Kv4 potassium channels (Zhou et al., 2008). They identified a specific AU-rich element in the 3′UTR of Kv4.3 that mediates AngII-dependent transcript destabilization, but showed that AngII-mediated binding of AUF1 had no effect on HuR to the Kv4.3 transcript (Zhou et al., 2008). AUF1 and HuR have been demonstrated to concurrently bind target mRNAs, with similar AU-rich target sequences, in a competitive and non-competitive manner (Lal et al., 2004).

AUF1 was also shown to bind to the 3′UTR of SERCA2a downstream of PKC activation in neonatal rat ventricular myocytes (Blum et al., 2005). Cardiac expression of SERCA2a is the result of alternative splicing that yields a differential exon inclusion and 3′UTR sequence between the SERCA2a isoform expressed in the heart and the more ubiquitously expressed SERCA2b isoform. The differential 3′UTR sequences between the two isoforms yields a difference in transcript stability that may contribute to the loss of SERCA2a expression in failing hearts, which exacerbates contractile dysfunction and contributes to pathological progression (Misquitta et al., 2002; Misquitta et al., 2005; Zhihao et al., 2020). Interestingly, the SERCA2a 3′UTR has five AU rich regions to which AUF1 and HuR are both predicted to bind, but Blum et al. were unable to detect HuR binding to SERCA2a in NRVMs (Blum et al., 2005).

However, both AUF1 and HuR have both been shown to exert post-transcriptional regulation via multiple regulatory mechanisms, including nuclear export, splicing, and translational control (Fan and Steitz, 1998; Doller et al., 2008; Izquierdo, 2008; Chang et al., 2014; Panda et al., 2014; Yoon et al., 2014). Similarly, HuR has been shown to act in concert with AUF1 to promote RNA destabilization (Chang et al., 2010). Another example of this was demonstrated in AUF1 and HuR independent regulation of Nrf2 expression, with HuR promoting nuclear export and AUF1 promoting RNA stability (Poganik et al., 2019). Nrf2 is a critical transcriptional mediator of cardiac I/R injury and response to oxidative stress (Chen and Maltagliati, 2018; Zang et al., 2020), but the potential significance of Nrf2 regulation by AUF1 or HuR has not yet been demonstrated in the failing heart. Similarly, AUF1 has been shown to regulate the expression of MEF2c, a key transcriptional regulator of cardiac hypertrophic signaling (Panda et al., 2014), but the importance of this interaction in pathological cardiac remodeling is unknown.

In addition to these tantalizing bits of data suggesting a prominent role for AUF1 in cardiomyocyte pathophysiology, there are also reports of AUF1 regulation of proliferation, migration, and cell senescence in skin and breast cancer associated fibroblasts that have gone unrealized in the cardiac field (Hendrayani et al., 2014; Wallis et al., 2015; Hendrayani et al., 2016; Yu et al., 2022).

#### Brain expressed X-linked protein 1 (Bex1)

Bex1 was identified to be increased in failing hearts where it associates with AU-rich mRNA targets, including many inflammatory gene products, as part of a larger ribonucleoprotein complex (Accornero et al., 2017). Cardiomyocyte-specific overexpression of Bex1 replicated a hypertrophic cardiomyopathy phenotype, while whole body deletion of Bex1 was protective against pressure overload-induced remodeling. Bex1 may play a more global role in muscle cell biology as the first report of Bex1-deficient mice showed an impairment in exercise performance and skeletal muscle regeneration (Koo et al., 2007). Interestingly, Bex1 may also bind to double stranded RNA and confer an antiviral role. Martens et al. recently showed that Bex1 plays a protective role against viral myocarditis and limits viral replication in both cardiomyocytes and fibroblasts (Martens et al., 2022).

Additional RBPs have been suggested to play a role in pathological remodeling through modulation of RNA stability, with much less known about the extent of their mechanisms or functional importance. For example, fused in sarcoma (FUS) is a ubiquitous and versatile protein involved in several cellular processes like DNA repair, gene transcription, oxidative stress, mitochondrial damage and cell apoptosis (Deng et al., 2015; Suzuki and Matsuoka, 2015; Bozzo et al., 2017; Singatulina et al., 2019; Birsa et al., 2020), and has been implicated in regulation of cardiomyocyte apoptosis in myocardial infarction models (Wu et al., 2018). More recently, FUS expression was shown to be induced downstream of AngII in cardiac fibroblasts and play a role in myofibroblast activity, but the mechanism or RNA targets of FUS remain largely unknown (Wang et al., 2021). More recently, PTBP was shown to also mediate cardiac fibrosis by promoting fibroblast proliferation and collagen deposition (Chen et al., 2023). This work also suggested a PTBP-dependent reduction in stability of the transcriptional regulator Nur77, highlighting the fact that many RBPs may act through multiple mechanisms as PTBP has already been mentioned mediate post-transcriptional regulation via splicing, translation, and alternative polyadenylation.

### Translational control

Gene expression resulting from transcriptional regulation is the most widely studied as a fundamental phenomenon, but it is essential to acknowledge the contribution of translational regulation as an equally important mediator of protein expression. The translational regulation of cardiac gene expressions plays a key role in determining heart function and disease, as it is influenced by natural genetic variation, which could lead to inefficient translation termination. Findings from genome-wide RNA sequencing and ribosome profiling have shown that a large number of cardiac genes carry distinct signatures in 3′UTR variation, RNA-binding protein motifs and miRNA expression which are associated with translational regulation of gene expression (Schafer et al., 2015). RBPs commonly mediate RNA regulation at the translational level either to promote activation or, more commonly, repression of the initiation of translation (Abaza and Gebauer, 2008). The mechanism of action of RBPs on translation initiation occurs through competing with ribosome binding, modification of the RNA structure to prevent its ribosomal recognition, or via direct RBP complex-mediated protein-protein interaction with the ribosomes themselves (Babitzke et al., 2009). One of the major RBP families known to regulate translation is the CUG-BP, Elav-like family (CELF) proteins. RBPs like CELF have dual roles to play during RNA processing events in development and during disease pathogenesis either as a cause or consequence. However, CELF proteins have versatile mechanisms of action; in the nucleus, they can mediate alternative splicing through exon exclusion, and in the cytoplasm, they can also bind to the 3′ UTR of target mRNA and control the translation of stability (Brinegar and Cooper, 2016).

The multifunctional role of RBPs was recently highlighted in a comprehensive ribo-seq and RNAseq profiling of human hearts that identified 21 RBPs which control both mRNA abundance and translational efficiency (Schneider-Lunitz et al., 2021). These RBPs were further analyzed using published eCLIP and HITS-CLIP data, and G3BP1, PUM1, DDX3X, DDX6, and ELF proteins were identified for their translational regulation role in the human left ventricle (Chothani et al., 2019; Luo et al., 2020; Schneider-Lunitz et al., 2021). These new results merit further investigation of translational control by RBPs as a key mediator of cardiac homeostasis and pathological remodeling.

### Novel roles of RBPs in HF

Our review of the mechanism of actions of RBPs that play a role in HF is mainly centered upon the most established mRNA modulatory actions like alternative splicing, mRNA degradation and translation. But newer investigations have shed light on the diverseness in their functionality in cardiovascular pathologies that extends beyond these mechanisms. As mentioned earlier RBPs can operate independently or in tandem with miRNAs, lncRNAs or circular RNAs and facilitate their actions to manifest effects on the heart. It’s also possible that RBPs may contribute to the generation of novel microproteins via stabilization, localization, or translational regulation of short ORFs identified within lncRNAs and circRNAs (Makarewich and Olson, 2017; van Heesch et al., 2019).

Studies conducted over the last decade have demonstrated that the regulatory effects of RBPs extend to beyond linear RNA transcripts, as RBPs have also been implicated in the biogenesis, expression and transport of circRNAs (Li et al., 2018). Most studies to date have explored the actions of circRNA as RBP-sequestrants or sponges and present evidence of competitive interaction between linear and circular RNA for regulatory RNA (e.g., miRNA) and RBP binding. Future work certainly needs to be aimed at increasing our understanding of circRNA-mediated mechanisms and determine whether specific targeting of circRNA-RBP complexes may modulate the initiation or progression of cardiac hypertrophy and fibrosis.

RBPs have also been shown to aid in the regulatory activities of mitochondrial non-coding RNA (ncRNA) and miRNAs also known as mitomiRs, by controlling their translocation from nucleus to mitochondria, by employing translocase-based sorting and assembly machinery (Wiedemann and Pfanner, 2017). RBPs execute this by associating themselves with a cytoplasmic multiprotein RNA-induced silencing complex (RISC), that consists of RBPs including protein kinase RNA activator, transactivation response RNA-binding protein (TRBP) and Dicer, which process pre-microRNAs into mature microRNAs. This overall mitochondrial regulation mediated by RISC has implications in HF owing to the energy dependence of cardiomyocytes on mitochondrial ATP (Jusic et al., 2020). Moreover, RBP contribution has also been documented in the active binding and transport of circulating cardiac miRNAs released by cardiac fibroblasts to be packaged into EVs, which in turn act as paracrine signaling mediators of cardiomyocyte hypertrophy (Tian et al., 2021).

In addition to transcriptional regulation, mRNA processing, and alternative processing, a cell can attain molecular complexity through post-transcriptional modification of RNA bases and gene editing. Through this cellular process RNA nucleotides in cell transcripts get altered in their sequence compared to the parent DNA they transcribed from (Gott and Emeson, 2000; Washburn and Hundley, 2016). The most well studied type of gene type of gene editing is the adenosine to inosine (A-I) editing in the dsRNA substrates, catalyzed by adenosine deaminase acting on RNA (ADAR) enzymes (Nishikura, 2010). The specific significance of A-I editing in growth and development, health and disease, various physiological processes, and relationship with RBPs has been reviewed elsewhere (Washburn and Hundley, 2016; Quinones-Valdez, 2019). ADARs are synonymous with RBPs and are either defined as a type of RBPs or known to act like RBPs, but additional RBPs influence ADAR-mediated gene editing by modifying certain RBP-binding sites to enhance editing or by altering the availability of dsRNA to suppress editing (Deffit, S. N., & Hundley, 2016). In the human heart, A-I editing is the predominant mechanism of gene editing, which is severely reduced during HF, accompanied by repressed ADAR2 expression and increased circRNA levels. Moreover, the potential importance of ADAR1 to cardiac remodeling was shown through augmented ventricular remodeling, cardiac dysfunction, unfolded protein response, and reduced miRNA expression following cardiomyocyte specific deletion of ADAR1 in adult mice (El Azzouzi et al., 2020).

RBPs also influence distinct post-transcriptional RNA modifications such as the N6-methyladenosine (m6A) methylation of mRNAs, rRNAs, tRNAs, long non-coding RNAs and microRNAs, that are vital for RNA splicing, transport, stability, and translation at the post-transcriptional level. Interestingly, cardiac hypertrophy and HF display aberrations in the m6A methylation of transcripts, thereby they can act as significant targets for deriving therapeutic strategies for managing CVD. This has also been further emphasized through the control of cardiac homeostasis and hypertrophy by The N^6^-methyladenosine mRNA methylase (METTL3) (Dorn et al., 2019). The cardiomyocyte RNA interactome in failing hearts was shown to contain 29 RBPs annotated for modifications like 5-methylcytosine, *N* ^6^-methyladenosine and pseudouridine modifications, and adenosine-to-inosine editing (Liao et al., 2016; Hentze et al., 2018). m6A has been implicated to be an important target as repressed m6A levels have shown to attenuate fibrosis via altered RNA splicing, translation and degradation (Mathiyalagan et al., 2019; Chen et al., 2021).

Some examples of RBPs that recognize m6A-modified mRNA are YT521-B homology (YTH), heterogenous nuclear ribonucleoprotein A2/B1 (HNRNPA2B1) and insulin-like growth factor 2 mRNA-binding protein (IGF2BP) domain (Qin et al., 2020; Fan and Hu, 2022). Heterogenous nuclear ribonucleoprotein A2/B1 (HNRNPA2B1) is also suggested to regulate cardiac homeostasis and hypertrophy via N6 methyladenosine mRNA methylase (METTL3) and m6A switch recognition and alternative splicing events (Qin et al., 2020; Fan and Hu, 2022). RBPs also contribute to the regulation of RNA m5C methylation, dysregulation of which is closely associated with CVD (Balachander et al., 2023).

## Conclusion

When cells of the myocardium are stressed or injured, they often initiate a functional remodeling of their transcriptome in order to minimize tissue damage and maintain cardiac structure and function. RBPs play a critical post-transcriptional regulatory role in this transcriptomic remodeling and their expression and activity are tightly coupled with dynamic changes in gene expression patterns that drive the pathophysiological response in cardiac remodeling (Table 1). As our understanding of the human genome has developed significantly, we have gained an increasingly comprehensive understanding of both protein-coding RNA transcripts and non-protein-coding RNA transcripts, as well as the RBPs that interact with them and coordinate their splicing, stability, and translation.

**TABLE 1:** An overview of the major cardiac RBPs that have been demonstrated to regulate pathological cardiac remodeling.

RNA-binding protein	Primary role in cardiac remodeling	Mechanism of action/target mRNA	Key references
RBM20	Deletion promotes contractile dysfunction and HF	Alternative splicing (titin, CamkII, RyR2, PDZ, Lim domain 5, PLN, Nebl, Ablim1, Enah)	Guo et al. (2012); Schneider et al. (2020)
RBM24	Overexpression promotes fibrosis	TgfβR1 and TgfβR2 expression	van den Hoogenhof et al. (2018); Liu et al. (2019)
Rbfox1/2/3	Decreased in TAC model. Deletion sufficient to induce contractile dysfunction and HF	Widespread alternative splicing (MEF2, CaV1.2 L-type calcium channels)	Wei et al. (2015); Gao et al. (2016)
MBNL	Increased following MI; Fibroblast activity	Splicing regulator (Calcineurin, SRF, p38)	Davis et al. (2015); Stempien-Otero et al. (2016); Bugg et al. (2022)
CELF1	CELF disruption promotes cardiac hypertrophy and fibrosis	Alternative splicing, mRNA stability, and translation (dependent on cellular location)	Ladd et al. (2005a); Ladd et al. (2005b);
HuR	Increased in failing hearts; Inhibition ameliorates pathological remodeling; Independently promotes myocyte hypertrophy and fibroblast activity	RNA stabilization (β2-AR, TGFß, p53, Wisp1)	Krishnamurthy et al. (2010); Slone et al. (2016); Green et al. (2019); Green et al. (2023)
PTBP	Cardiac development; Fibroblast proliferation and collagen deposition	Translation (Apaf-1, Caspase-3); Splicing (β-arrestin-1; Stability (Nur77)	Zhang et al. (2009); Liu et al. (2023); Chen et al. (2023)
AUF1	Increased in failing hearts	ARE-mediated RNA degradation of β1-AR, Kv4.3, MEF2c, and SERCA2a	Pende et al. (1996); Blum et al. (2005); Zhou et al. (2008); Panda et al. (2014)
Bex1	Increased in failing hearts. Deletion protective against pressure overload remodeling; Overexpression in myocytes induces hypertrophy; Protective in viral myocarditis	Stabilizes TNF α mRNA in cardiomyocytes	Accornero et al. (2017); Martens et al. (2022)
FUS	Cardiomyocyte apoptosis; Fibroblast activity	Precise mechanisms unknown	Wu et al. (2018); Garikipati et al. (2019); Wang et al. (2021)
SRSFs	Regulate cardiac contraction, systolic HF and calcium handling	Alternative splicing (mTOR, triadin)	Ortiz-Sanchez et al. (2019); Feng et al. (2019)
RBPMS	Myofibrillar organization and calcium handling	Alternative splicing (titin, Pdlim5, nexilin)	Akerberg et al. (2022); Gan et al. (2022) & (2023)
QKI	Cardiomyocyte contractility	Alternative splicing (sarcomere, cytoskeletal, and calcium-handling genes)	Kelaini et al. (2021); Cornelius et al. (2022)
HNRNPA2B1	Controls cardiac homeostasis and hypertrophy	Involves N6 methyladenosine mRNA methylase (METTL3) and m6A switch	Qin et al. (2020); Fan & Hu. (2022)

The interaction of RBPs with target mRNAs are often regulated by post-translational modifications, cofactor binding and protein-protein interactions. RBPs have historically been considered to be an undruggable class of proteins due to their high number of potential RNA targets, relatively weak protein-RNA binding interactions, and the resulting difficulties in targeting specific RBP-target RNA interactions. However, there have been recent developments in RNA-based therapeutics that act in an inhibitory manner by sponging specific RNA-binding proteins and reducing their binding and regulation of endogenous target RNAs (Schreiner et al., 2020; Wang and Liu, 2020). For example, AAV9-mediated expression of the circRNA circFndc3b was found to reduce pathological cardiac remodeling in a mouse myocardial infarction model, and the proposed mechanism is via circRNA binding and sponging of the RBP FUS to reduce FUS binding to endogenous targets (Garikipati et al., 2019). Similar approaches to target specific miRNAs via regRNAs, lncRNAs and circRNAs sponges have also been studied in oncology models, but the application of these approaches to the heart are still in very early stages (Zhang et al., 2019; Gomes et al., 2020; Jiang et al., 2021). As our mechanistic understanding of RBP-RNA interactions and their functional consequences to cardiac physiology, so too should the optimism that RBP-based therapeutics may hold promise as novel approaches to pathological cardiac remodeling.
